# Clinical and economic burden of major lower-limb amputations attributable to peripheral artery disease in Italy: A nationwide analysis

**DOI:** 10.1371/journal.pone.0361853

**Published:** 2026-11-02

**Authors:** Matteo Scortichini, Mauro Gargiulo, Marco Manzi, Luigi Uccioli, Paolo Sciattella

**Affiliations:** 1 Economic Evaluation and Health Technology Assessment, Centre for Economic and International Studies, Faculty of Economics, University of Rome Tor Vergata, Rome, Italy; 2 Vascular Surgery, Department of Medical and Surgical Sciences (DIMEC), University of Bologna, Bologna, Italy; 3 Vascular Surgery Unit, IRCCS Sant'Orsola, Azienda Ospedaliero-Universitaria, Bologna, Italy; 4 Interventional Radiology Unit, Foot & Ankle Clinic Policlinico Abano Terme, Abano Terme, Italy; 5 UOC Diabetes and Endocrinology, CTO Hospital, Department of Surgical Science, University of Rome “Tor Vergata”, Rome, Italy; Hiroshima University: Hiroshima Daigaku, JAPAN

## Abstract

Peripheral artery disease (PAD) represents a major cause of non-traumatic lower-limb amputation and is associated with substantial morbidity, mortality, and healthcare costs. Despite advances in medical and revascularization therapies, major amputations remain frequent, and comprehensive nationwide estimates of their clinical and hospital-related economic burden within the Italian National Health Service (SSN) are limited. The objectives of this study were to estimate the hospital-related economic burden of major lower-limb amputations attributable to PAD in Italy and to assess revascularization patterns and in-hospital mortality within the SSN. We conducted a nationwide observational study using the Italian National Hospital Discharge Database. Acute hospital discharges between 2015 and 2021 with a major lower-limb amputation and Diagnosis Related Group 113 were identified. Inpatient volumes, length of stay, and costs were estimated by amputation level. A longitudinal cohort of patients amputated between 2015 and 2017 was followed for two years before and after the index hospitalization to assess prior revascularization and in-hospital mortality using age- and sex-adjusted Cox models. A total of 26,417 PAD-attributable major amputations were identified, 75.6% of which were above-knee procedures. Cumulative inpatient expenditure reached €294 million, corresponding to over 511,000 hospital days. Only 46.0% of patients underwent revascularization before amputation. In-hospital mortality increased steeply within the first six months post-amputation and remained higher after above-knee procedures and among patients with diabetes, reaching 16–25% at 180 days and 23–34% at two years. Major lower-limb amputation remains a severe and costly outcome of PAD in Italy. Improved implementation of guideline-recommended PAD management is needed to reduce avoidable amputations and their economic burden on the SSN.

## Introduction

Chronic peripheral obstructive arterial disease is a progressive disorder of the lower extremities caused predominantly by atherosclerosis and characterized by obstruction of peripheral arteries, leading to ischemia, impaired function, and increased cardiovascular mortality [1]. Peripheral artery disease (PAD) is considered an indicator of widespread atherosclerosis and shares risk factors (age, smoking, hypertension, dyslipidemia) with coronary and cerebrovascular diseases [2]. Patients may develop intermittent claudication (exercise-induced leg pain) and, in advanced stages, critical limb threatening ischemia (CLTI) with rest pain, ulcers, or gangrene [3,4]. PAD currently affects over 200 million individuals globally, and its prevalence is projected to increase by nearly 50% by the year 2045 [5].

Despite its high prevalence, PAD often goes undiagnosed and untreated, as individuals in the early stages typically do not report, or may not experience, typical claudication symptoms such as leg pain [6]. The PANDORA study, conducted on a large European cohort of 10,287 individuals at non-high cardiovascular risk, assessed the prevalence of asymptomatic PAD: although the study is outdated, the results revealed considerable variability across countries, with Italy showing a notably high prevalence rate of 22.9%, second only to Greece (28.0%), highlighting a significant burden of undiagnosed PAD even in populations considered at lower cardiovascular risk [7].

Diabetes mellitus is one of the major risk factors for PAD. People with diabetes are roughly twice as likely to develop PAD as those without diabetes [8]. The combination of diabetes and PAD has a synergistic effect on limb risk: these patients have significantly higher rates of non-healing foot ulcers, infections, and limb loss than those with PAD alone [9,10]. In diabetic cohorts, PAD prevalence is higher: one European study of patients over age 70 with diabetes found that around 61% had clinical PAD (history of revascularization or ulcer) and 71% had PAD by ankle–brachial index criteria [11]. Incidence data likewise show elevated risk: for example, a long-term study found that nearly 16% of people with diabetes and no previous history of cardiovascular diseases (CVD) developed PAD over 5.5 years [12].

PAD represents one of the main causes of non-traumatic lower-limb amputation. Between 2000 and 2013, 21 countries of the Organisation for Economic Co-operation and Development (OECD) recorded a significant reduction in diabetes-related major lower-limb amputations, with an average decrease of 30.6% in the general population and 29.8% among individuals with diabetes [13]. Despite these overall improvements, the burden of non-traumatic lower-limb amputation remains disproportionately high among patients with diabetes, and this is particularly concerning given the aging population and the consequent rise in diabetes prevalence [5,14]. Amputation rates have remained stable or even increased in specific diabetic subgroups, and individuals with diabetes have a four- to five-fold higher risk of lower-limb amputation compared with those without diabetes [15,16]. A large retrospective analysis based on national Italian data further confirmed this disproportionate burden, showing that patients with diabetes have an approximately ten-fold higher risk of lower-limb amputation compared with non-diabetic patients [17].

Major lower-limb amputations impart a heavy economic toll on healthcare systems and patients. Evidence from the US REACH registry shows that patients with a history of peripheral interventions represent the subgroup with the highest hospitalization costs. Over a 2-year follow-up, mean cumulative hospitalization costs per patient exceeded $10,000 among individuals with prior lower-limb amputation or revascularization, compared with approximately $7,000 in patients with asymptomatic PAD or claudication alone. Importantly, a history of lower-limb amputation was associated with higher rates of subsequent hospitalizations and repeat procedures [18]. In a large U.S. registry focused on veterans with diabetes undergoing lower limb amputations, the median hospitalization costs associated with major lower-limb amputations were estimated at $48,885 for below-knee and $55,371 for above-knee procedures in 2010, with total annual expenditures exceeding $200 million [19].

In addition to the substantial economic burden, major lower-limb amputation is associated with a markedly poor prognosis, with studies reporting 30–50% mortality within 3–5 years [4,10,20]. Perioperative mortality increases significantly with the level of amputation, ranging from approximately 5–10% for below-knee procedures to 15–20% for above-knee amputations [21]. An Italian population-based study in the Lazio region found that among patients with diabetes undergoing lower-extremity amputation, mortality reached 65% at four years after major amputation and 45% after minor amputation, highlighting a substantial long-term mortality risk and the impact of factors such as age, renal disease, and depression [22].

Management of PAD relies on intensive control of modifiable risk factors, such as smoking, hypertension, dyslipidemia, and hyperglycemia, along with medical therapies including antiplatelet agents, statins, other lipid- or anticoagulant-based regimens to reduce cardiovascular and limb-related events [23]. Despite the proven benefits of these interventions, including supervised exercise programs and preventive foot care in diabetic patients, PAD remains widely underdiagnosed and undertreated, underscoring the need for improved adherence to clinical guidelines [10,24].

Revascularization (surgical bypass or endovascular angioplasty) is a key intervention for limb salvage in PAD, especially for CLTI. Current guidelines emphasize that patients with critical ischemia should undergo revascularization whenever feasible to restore perfusion and minimize amputation risk [4,25]. Nevertheless, recent evidence underscores that optimal outcomes in advanced PAD and CLTI are closely linked to timely referral and structured multidisciplinary fast-track pathways, which, although successfully implemented in selected Italian centers, are not yet uniformly available, suggesting the persistence of unmet needs in care organization and access at the national level [21].

The aim of this study is to analyze the clinical and economic burden of major lower-limb amputations in patients with PAD. Specifically, we sought to estimate the number of major amputations performed in this population, quantify the associated healthcare resource use and hospital-related expenditure borne by the Italian National Health Service (SSN), and assess in-hospital mortality rates following amputation. In addition, we evaluated the proportion of amputated patients who had previously undergone a revascularization procedure.

## Data and methods

### Data

We used the Italian National Hospital Discharge Database (Scheda di Dimissione Ospedaliera, SDO), which compiles records of all inpatient hospital discharges (HDs) from public and private hospitals nationwide. This administrative database includes detailed information on demographics, diagnoses, procedures, and outcomes for each hospitalization, together with an anonymous unique patient identifier (available only from 2013 to 2019). The database was accessed for the purposes of this study on January 29, 2025. The authors did not have access to information that could identify individual participants either during or after data collection, as all data were fully anonymized prior to access.

### Methods

#### Cross sectional analysis.

We identified all acute care HDs (ordinary inpatient stays) of residents in Italy during 2015–2021 that involved a major lower-limb amputation procedure potentially related to PAD. To identify all major lower-limb amputations, we selected all HDs with any of the following ICD-9-CM codes recorded as either the primary or secondary procedure:

- 84.13 Disarticulation of ankle
- 84.14 Amputation of ankle through malleoli of tibia and fibula
- 84.15 Other amputation below knee
- 84.16 Disarticulation of knee
- 84.17 Amputation above knee
- 84.18 Disarticulation of hip
- 84.19 Abdominopelvic amputation

To focus on amputations related to PAD, we only included HDs with Diagnosis Related Group (DRG) 113: “Amputation for circulatory system disorders except upper limb and toe with cc”.

To quantify the burden of major lower-limb amputations, we calculated the total number of HDs with these procedures for each year in the study period. We also estimated the percentage of HDs in which a revascularization procedure was performed concurrently with the amputation. Furthermore, we assessed healthcare resource utilization by measuring the total and mean length of hospital stay per HD, as well as the associated costs based on the DRG reimbursement tariffs attributed to each HD. All financial data were reported in euros and reflect the expenditures reimbursed by the Italian SSN. Results were stratified by level of amputation: below knee (ICD-9-CM 84.13–84.15) or above knee (84.16–84.19). Revascularization was identified by one of the following ICD-9-CM codes, either in principal or secondary procedure:

- 39.50 Angioplasty or atherectomy of other non-coronary vessel(s)
- 39.90 Insertion of non-drug-eluting peripheral vessel stent(s)
- 00.55 Insertion of drug-eluting peripheral vessel stent(s)
- 39.25 Aorta-iliac-femoral bypass
- 39.29 Other (peripheral) vascular shunt or bypass
- 38.18 Endarterectomy, lower limb arteries
- 38.48 Resection of vessel with replacement, lower limb arteries
- 38.38 Resection of vessel with anastomosis, lower limb arteries
- 05.23 Lumbar sympathectomy

In cases where multiple amputation procedures were coded in the same HD, the hospitalization was categorized by the most severe amputation level (for example, if both a below-knee and an above-knee amputation were performed, the case was classified as an above-knee amputation).

### Longitudinal analysis

All patients with a major lower-limb amputation between 2015 and 2017 were enrolled, first HD during the enrollment period with a procedure of amputation was considered as the index hospitalization. For each patient, we reconstructed the clinical history during the two years preceding the index hospitalization (baseline period), focusing on HDs recorded across Italian healthcare facilities. A descriptive analysis was conducted to characterize the age at the index hospitalization and sex distribution of the selected cohort. In addition, we reported the proportion of patients who, during the baseline period, underwent at least one lower-limb revascularization procedure and/or a minor amputation, defined as ICD-9-CM 84.11 (amputation of toe) or 84.12 (amputation through foot). We also calculated the percentage of patients who had a diagnosis of gangrene during the baseline period. Gangrene was identified by the following ICD-9-CM codes: 040.0 (gas gangrene), 440.24 (atherosclerosis of the extremities with gangrene), and 785.4 (gangrene, unspecified).

Hospital resource use during the baseline was assessed by summing the total number of inpatient days per patient across all admissions in Italian hospitals. Moreover, we estimated the distribution of time from the last revascularization procedure (if any) during the baseline period to the index amputation.

To assess post-amputation prognosis, we applied an age- and sex-adjusted Cox proportional hazards model [26] to estimate in-hospital mortality within two years following the index hospitalization (follow-up period). The enrollment period was restricted to 2015–2017 to ensure a minimum two-year follow-up for all individuals. Data from 2020–2021 were excluded from the longitudinal analysis because anonymized patient identifiers were not available in the SDO dataset for those years.

Finally, we estimated the proportion of in-hospital deaths that had a primary or secondary diagnosis related to CVD (ICD-9-CM codes 390–459). All analyses were stratified by the level of amputation (below knee, above knee) and by the presence of a diabetes diagnosis (ICD-9-CM 250.XX) during the baseline period.

## Results

### Cross sectional analysis

Between 2015 and 2021, a total of 35,099 acute HDs in ordinary regimen with major lower-limb amputation were recorded across Italy; of these, 26,417 (75.3%) were potentially attributable to PAD. As shown in Fig 1, the annual number of HDs for above-knee amputations (AKAs) consistently exceeded those for below-knee amputations (BKAs) throughout the study period (75.6% vs 24.4%). AKAs decreased from 3,060 in 2015 to 2,532 in 2020, with a slight increase observed in 2021 (2,622). BKAs showed a slight downward trend over the study period, declining to 859 cases in 2021 after peaking at 1,011 in 2016.

**Fig 1:**
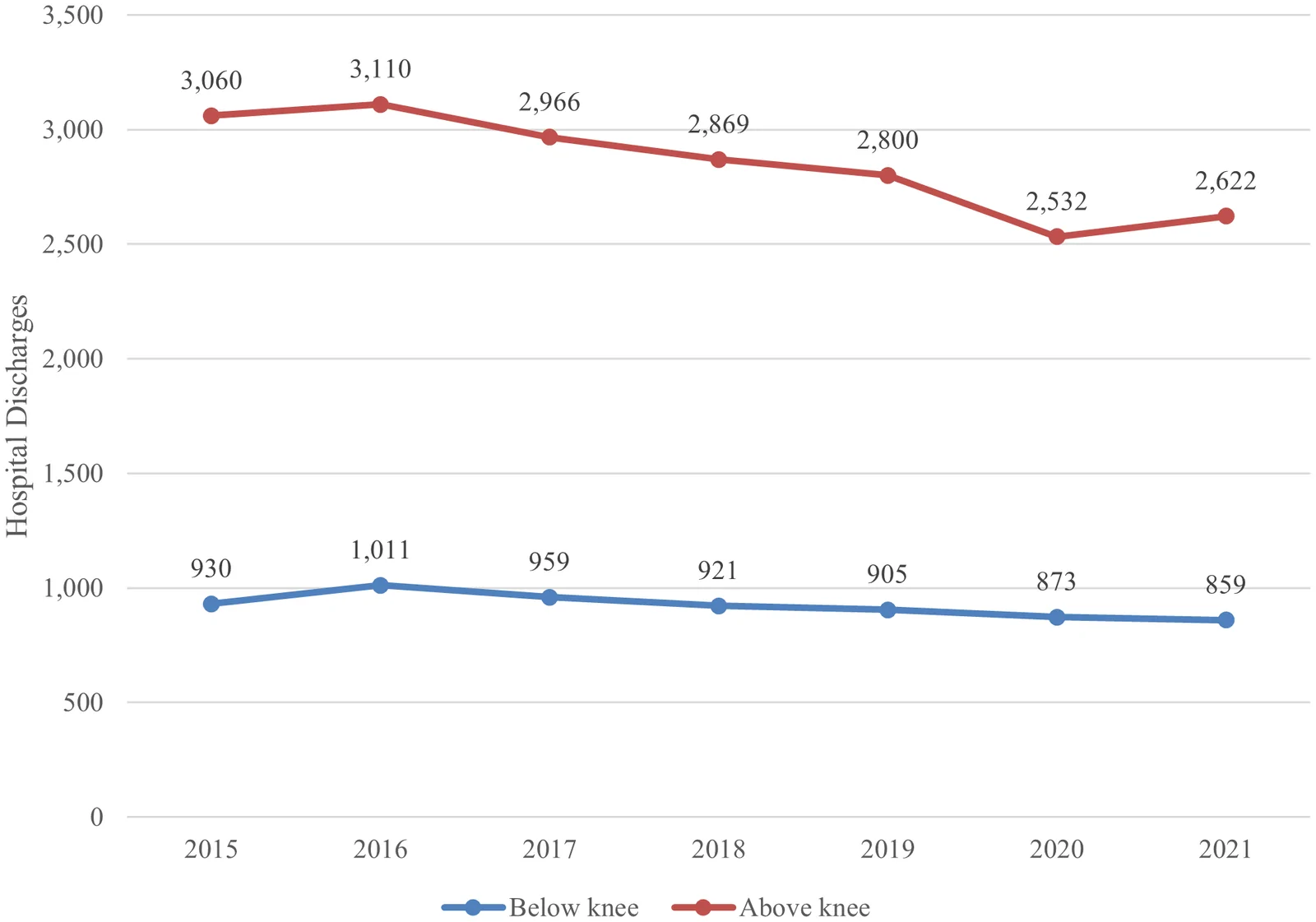
Annual time series of hospital discharges with a procedure of Major Amputation of lower limb, by amputation level, Italy 2015-2021.

Fig 2 illustrates the proportion of HDs involving a revascularization procedure performed during the same admission as the major amputation. For BKAs, this percentage increased steadily from 16.5% in 2015 to a peak of 23.4% in 2020, slightly declining to 22.7% in 2021. In contrast, revascularization rates associated with AKAs remained consistently lower and relatively stable throughout the study period, though a modest upward trend was observed beginning in 2019 (13.5%), peaking in 2020 (14.7%), and slightly declining in 2021 (13.7%).

**Fig 2:**
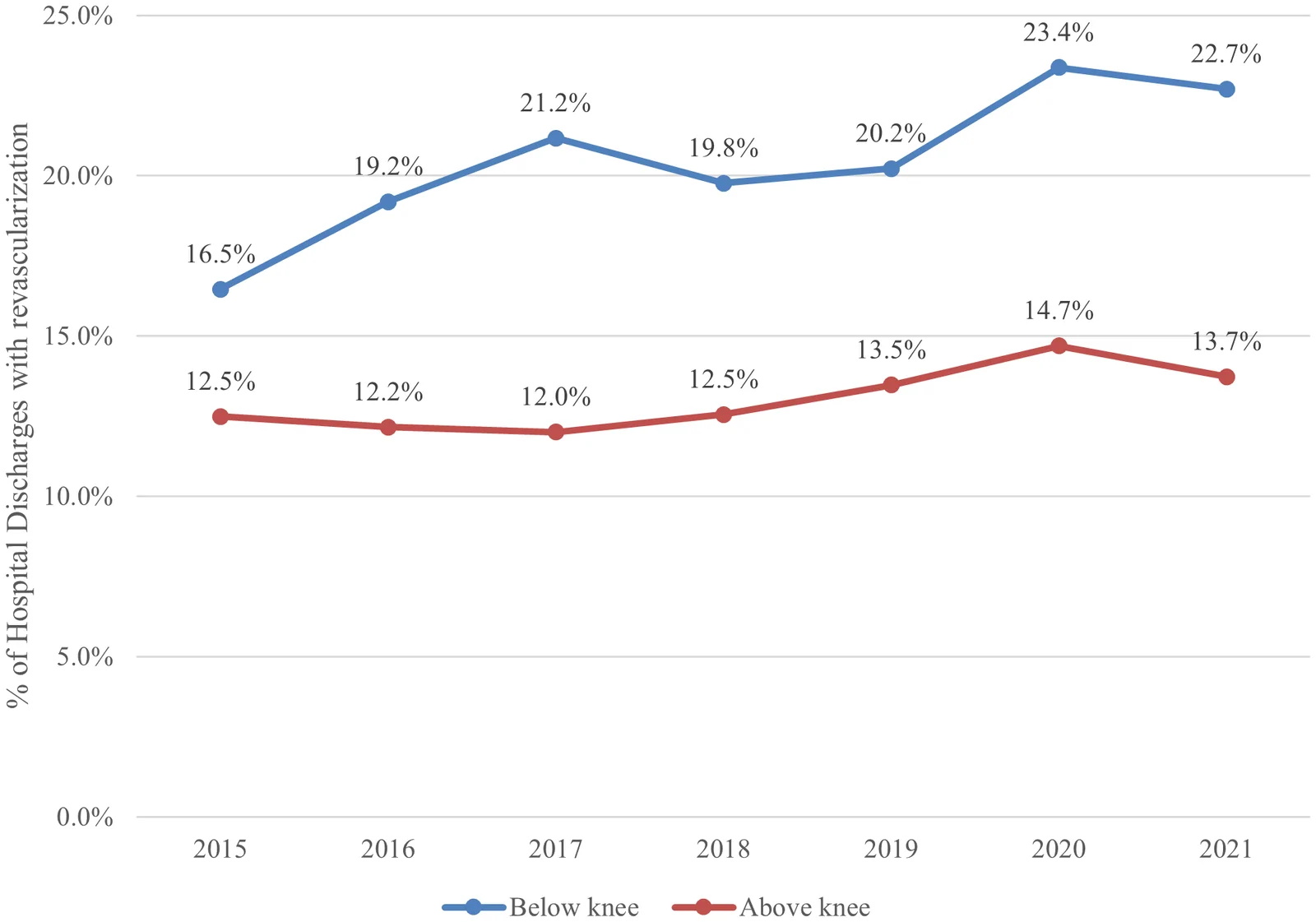
Annual time series of the proportion (%) of revascularization in hospital discharges with a procedure of Major Amputation of lower limb, by amputation level, Italy 2015-2021.

The economic and healthcare burden associated with major amputations is depicted in Fig 3. The cumulative hospital expenditure borne by the SSN over the seven-year period was €294 million, with AKAs accounting for €221.8 million and BKAs for €72.2 million. The total number of inpatient days associated with major amputations was 511,790, of which 372,145 days were for AKAs and 139,645 for BKAs. The mean cost per HD was similar between the two groups: €11,115 for AKAs and €11,182 for BKAs. However, BKAs were associated with a longer mean length of stay (LOS, 22 days) compared to AKAs (19 days), indicating a greater intensity of inpatient care in the former group.

**Fig 3:**
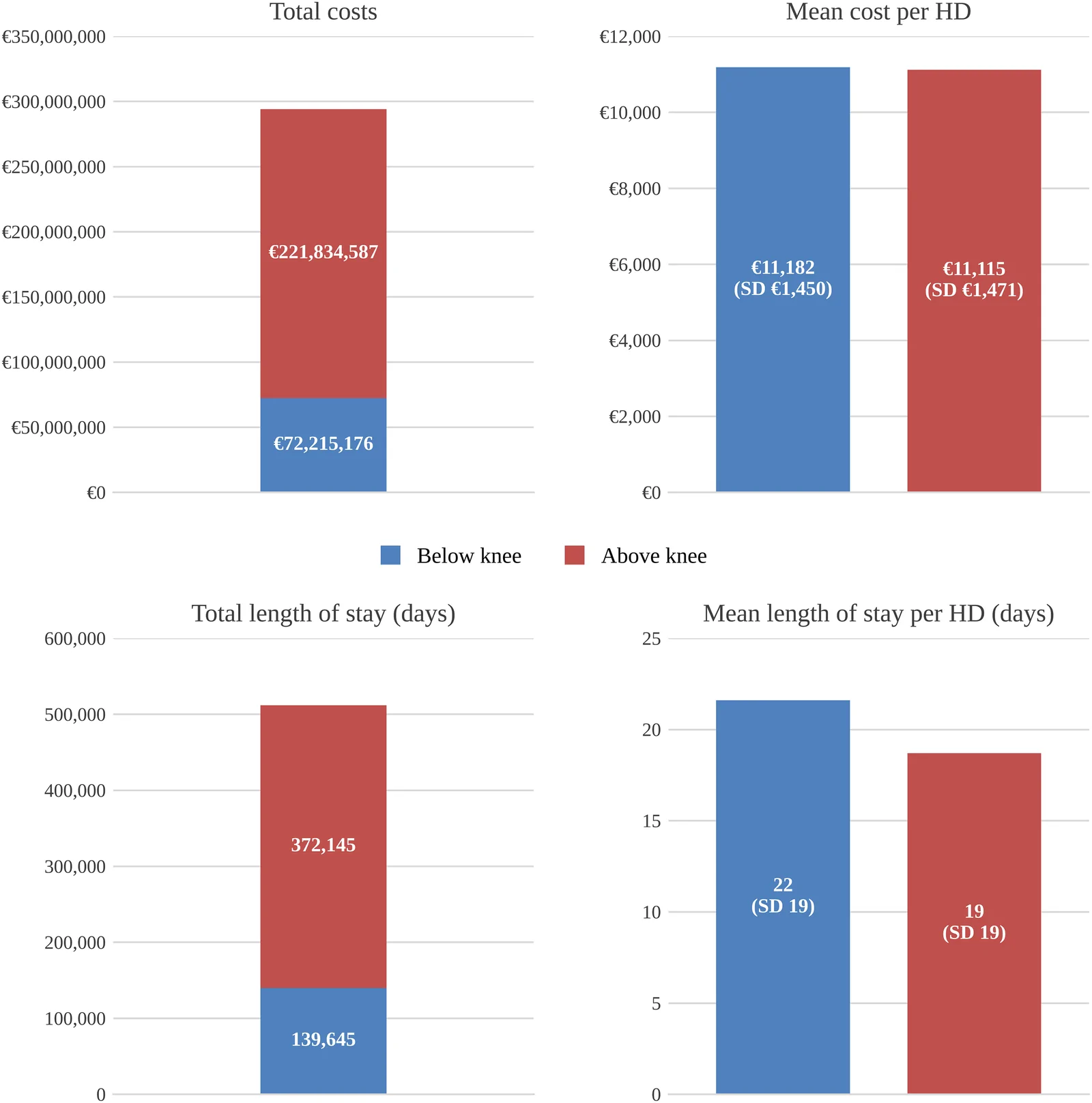
Costs and Length of Stay related to hospital discharges with a procedure of Major Amputation of lower limb, by amputation level, Italy 2015-2021.

### Longitudinal analysis

Between 2015 and 2017, a total of 11,217 patients undergoing a major lower-limb amputation were included in the longitudinal cohort. Overall, 61.1% were male and 56.4% had a documented diagnosis of diabetes (Table 1). Patients undergoing AKAs were older on average (mean age 80.1 ± 10.9 years for non-diabetics and 76.1 ± 10.1 years for diabetics) compared with those undergoing BKAs (72.5 ± 14.5 and 70.2 ± 11.2 years, respectively). The proportion of males was higher among patients with diabetes, particularly in the BKA group, where 72.7% were male compared with 61.7% in the AKA diabetic subgroup. Age distribution showed wide heterogeneity, with standard deviations ranging from 10 to 15 years, indicating markedly high variability in patient age at the time of amputation.

**Table 1:** Demographic characteristics of patients with a hospital discharge with a procedure of major amputation of lower limb, stratified by amputation level and diabetes status, Italy 2015-2017.

Amputation	Diagnosis of diabetes	Patients	% males	Age	
				Mean	SD
Above knee	No	4,155	54.4%	80.1	10.9
	Yes	4,274	61.7%	76.1	10.1
	Total	8,429	58.1%	78.1	10.7
Below knee	No	736	62.2%	72.5	14.5
	Yes	2,052	72.7%	70.2	11.2
	Total	2,788	69.9%	70.8	12.2
Total	No	4,891	55.6%	79.0	11.8
	Yes	6,326	65.3%	74.2	10.8
	Total	11,217	61.1%	76.3	11.5

In the two years preceding the index hospitalization, revascularization procedures (either surgery or endovascular) were more frequently observed in patients undergoing BKAs (57.5%) than in those with AKAs (42.2%), with consistently higher rates among diabetic patients. Among BKA patients with diabetes, 32.8% underwent revascularization in combination with a minor amputation, compared to 19.0% among non-diabetics. The diagnosis of gangrene during the baseline period was highly prevalent across all subgroups, exceeding 84% and reaching 93.2% among diabetic BKA patients. The average cumulative length of hospital stay during the baseline period was substantially higher in diabetic patients (39.6 days for AKAs and 40.4 days for BKAs) compared to non-diabetics (24.9 and 30.5 days, respectively), indicating a significantly greater use of hospital resources prior to amputation in the diabetic population. On average, the mean LOS per patient was equal to 33.7 days (Table 2). Among patients who underwent at least one revascularization during the baseline period, the mean number of revascularization procedures per patient ranged from 1.6 in AKA patients to 1.8 in BKA patients. This pattern was consistent across diabetes strata, with slightly higher mean values in diabetic than in non-diabetic patients (1.7–1.8 vs 1.6).

**Table 2:** Healthcare resource utilization in the two years preceding index hospitalization for major lower-limb amputation, stratified by amputation level and diabetes status.

Amputation	Diagnosis of diabetes	Patients	Patients with revascularization during baseline			Mean number of revascularizations per patient with at least one revascularization during baseline	Patients with gangrene diagnosis during baseline	Mean length of stay in ordinary regimen during baseline (days)
			Total	During index event	With minor amputation			
Above knee	No	4,155	38.8%	15.1%	6.4%	1.6	84.4%	24.9
	Yes	4,274	45.4%	10.5%	16.9%	1.7	92.6%	39.6
	Total	8,429	42.2%	12.7%	11.7%	1.6	88.6%	32.3
Below knee	No	736	58.3%	22.8%	19.0%	1.7	88.2%	30.5
	Yes	2,052	57.3%	18.2%	32.8%	1.8	93.2%	40.4
	Total	2,788	57.5%	19.4%	29.2%	1.8	91.9%	37.8
Total	No	4,891	41.8%	16.2%	8.3%	1.6	85.0%	25.7
	Yes	6,326	49.3%	13.0%	22.1%	1.8	92.8%	39.8
	Total	11,217	46.0%	14.4%	16.1%	1.7	89.4%	33.7

Fig 4 shows the cumulative distribution of time from the last revascularization procedure during the baseline period to the index hospitalization, stratified by amputation level. Among patients undergoing AKA who had at least one revascularization during baseline, 30% underwent revascularization during the index hospitalization (day 0), and 77% had been revascularized within 180 days prior to the index event. The cumulative proportion reached 50% at 57 days and 75% at 162 days before the index hospitalization.

**Fig 4:**
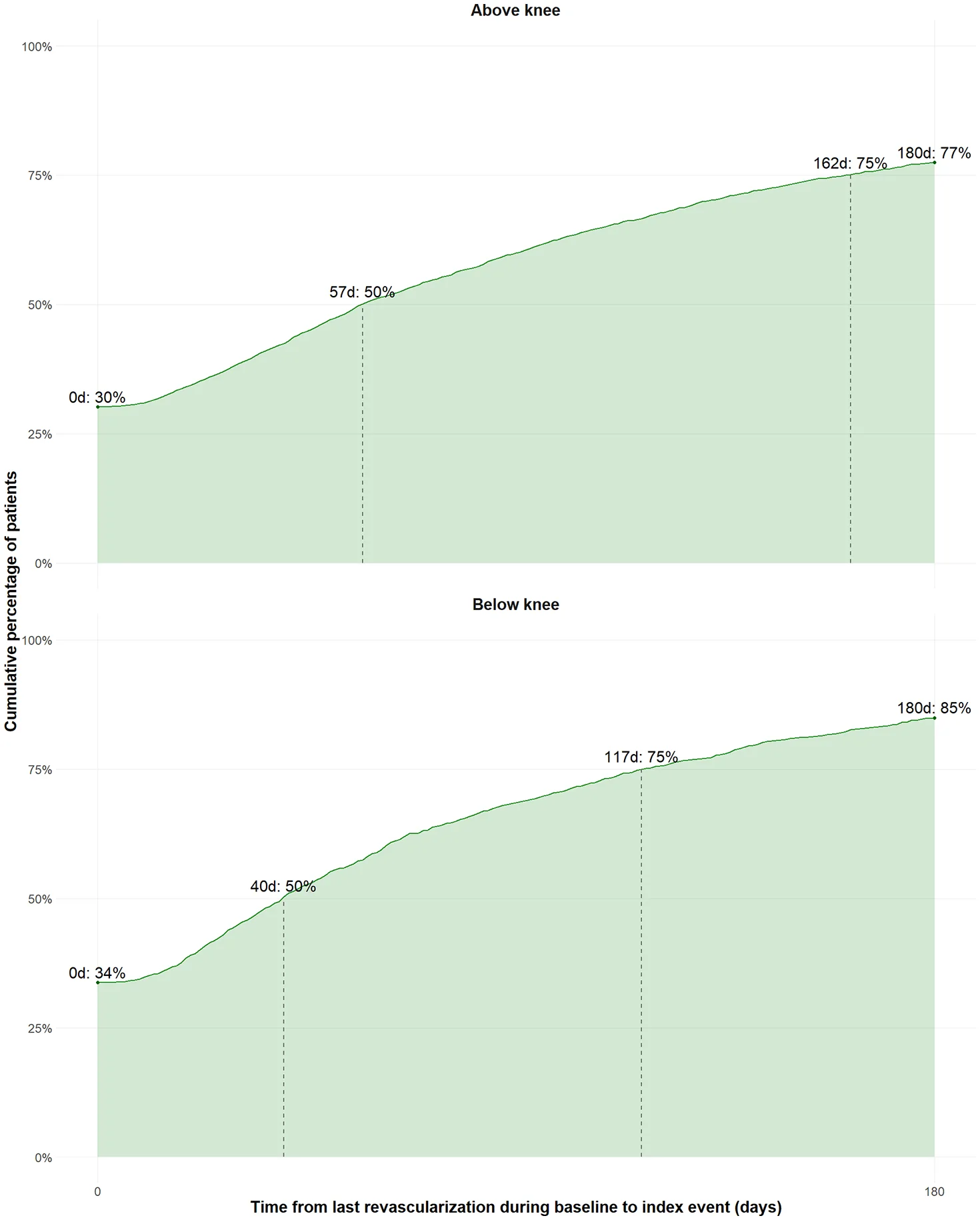
Cumulative distribution of time from last revascularization during baseline to index hospitalization in patients with revascularization.

Among BKA patients, 34% underwent revascularization during the index hospitalization, and 85% had been revascularized within 180 days prior to the index event. The cumulative distribution reached 50% at 40 days and 75% at 117 days before the index hospitalization, indicating an overall earlier timing of revascularization procedures in this group.

Fig 5 presents age and sex-adjusted Cox model estimates of in-hospital mortality over the two-year period following the index major lower-limb amputation, stratified by amputation level and diabetes status. Mortality risk was consistently higher among patients undergoing AKAs compared to BKAs, and within each stratum, patients with diabetes had worse outcomes than their non-diabetic counterparts. The adjusted Cox proportional hazards model showed a significantly lower hazard of in-hospital mortality for BKA compared with AKA patients (HR 0.71; 95% CI 0.65–0.78), after adjustment for age and sex. At 730 days of follow-up, the estimated in-hospital mortality reached 34% in the AKA-diabetes group, compared to 30% in the AKA-non-diabetes group. For BKA patients, mortality reached 25% among those with diabetes and 23% among those without. The steepest increase in mortality occurred within the first 6 months post-amputation across all groups, with 180 days mortality rates ranging from 16% to 25%. Interestingly, significant mortality rates were observed during index hospitalization, ranging from 8% (BKA-non-diabetes) to 13% (AKA-diabetes).

**Fig 5:**
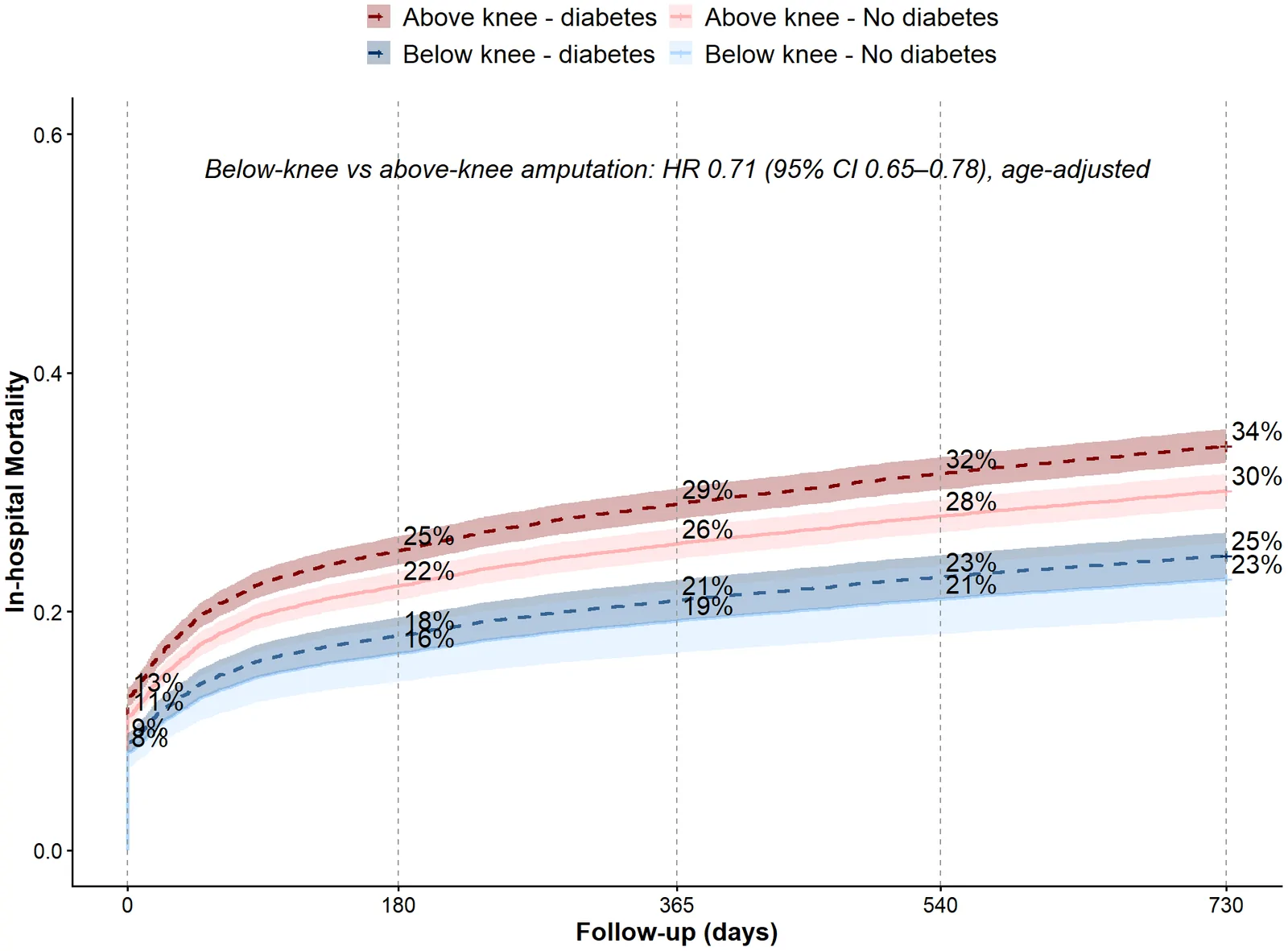
Age and sex-adjusted Cox estimates of in-hospital mortality in the two years following index major lower-limb amputation, stratified by amputation level and diabetes status.

Table 3 presents cardiovascular-related mortality during the index hospitalization and across the full two-year follow-up, stratified by amputation level and diabetes status. Overall, among the 1,281 patients who died during the index hospitalization, 72.8% had CVD listed as the principal diagnosis, and 92.6% had CVD listed as either a principal or secondary cause. Cardiovascular mortality during the index hospitalization was more frequent among patients without a diagnosis of diabetes, both in the AKA group (85.4% vs 62.9% in patients with a diagnosis of diabetes) and in BKA patients (85.1% vs 56.7%).

**Table 3:** Cardiovascular-related mortality during index hospitalization and 2-year follow-up by amputation level and diabetes status.

Amputation	Diagnosis of diabetes	Patients	Death during index hospitalization			Death during 2-year follow-up (index hospitalization included		
			Total	CVD (only principal diagnosis)	CVD (principal or secondary)	Total	CVD (only principal diagnosis)	CVD (principal or secondary)
Above knee	No	4,155	553	85.4%	96.0%	1,304	51.3%	80.7%
	Yes	4,274	561	62.9%	90.7%	1,451	43.6%	79.7%
	Total	8,429	1,114	74.1%	93.4%	2,755	47.2%	80.2%
Below knee	No	736	47	85.1%	93.6%	162	41.4%	74.7%
	Yes	2,052	120	56.7%	85.0%	482	34.9%	74.5%
	Total	2,788	167	64.7%	87.4%	644	36.5%	74.5%
Total	No	4,891	600	85.3%	95.8%	1,466	50.2%	80.0%
	Yes	6,326	681	61.8%	89.7%	1,933	41.4%	78.4%
	Total	11,217	1,281	72.8%	92.6%	3,399	45.2%	79.1%

During the two-year follow-up (including the index hospitalization), a total of 3,399 deaths were recorded. Of these, 45.2% had CVD as the principal diagnosis and 79.1% had CVD as either principal or secondary diagnosis. When only considering the principal diagnosis, cardiovascular mortality remained higher in patients without a diagnosis of diabetes, although the gap was narrower compared to the index hospitalization. Almost no difference was observed by diabetes diagnosis in the rate of cardiovascular mortality when considering both principal and secondary diagnosis, while rates were still higher in patients with AKA compared to BKA.

## Discussion

This study aimed to evaluate the clinical and economic burden of major lower-limb amputations among patients with PAD in Italy, by estimating the number of procedures performed, quantifying hospital resource use and SSN-related inpatient expenditure, assessing in-hospital mortality, and determining the proportion of amputated patients with a history of prior revascularization. Using the Italian SDO, we identified 35,099 acute ordinary-regimen HDs with a major lower-limb amputation between 2015 and 2021, of which 26,417 (75.3%) met our definition of being potentially attributable to PAD (DRG 113). This confirms that PAD-related major amputation remains a substantial public-health issue for the SSN, despite the long-term reductions in diabetes-related amputation rates reported across OECD countries (Italy included) in previous decades [13]. Importantly, these findings should be interpreted in the context of a steadily increasing prevalence of diabetes [5,14], particularly pronounced in Italy due to population ageing [27], which could sustain the future burden of PAD and diabetes-related limb complications despite improvements in vascular care.

Across the entire 2015–2021 period, AKAs accounted for 75.6% of PAD attributable hospital discharges, consistently exceeding BKAs. Although the absolute number of AKAs declined between 2015 and 2020, with a partial rebound in 2021, the overall distribution remained markedly skewed toward more proximal amputations. This persistent predominance of AKAs suggests that, among patients reaching the hospital threshold captured by DRG 113, a substantial proportion could still present with advanced disease severity, extensive tissue loss or infection, frailty, or unfavorable anatomical patterns that limit the feasibility or durability of limb-preserving strategies. However, beyond patient-level clinical factors, system-level determinants may also play a relevant role. International and European guidelines for the management of CLTI consistently recommend that, whenever a healable and durable stump can reasonably be achieved, preservation of the knee joint should be pursued because of its major implications for postoperative mobility, prosthetic use, and rehabilitation outcomes [4,28]. In this context, the persistently high proportion of AKAs observed in our analysis may partly reflect suboptimal patient pathways and heterogeneity in institutional capacity to manage PAD and CLTI across Italian hospitals, including delayed referral to specialized vascular centers, limited or uneven access to revascularization procedures, and variability in the availability of multidisciplinary limb-salvage teams. Such organizational factors may increase the likelihood that patients are admitted at a stage where transtibial amputation is no longer feasible or carries an unacceptably high risk of failure. Supporting this interpretation, a study showed that, in patients with CLTI, BKAs are associated with a substantially higher risk of wound complications compared with AKAs (odds ratio 4.13; 95% CI: 2.32–7.35), as well as greater post-acute healthcare utilization, including higher hospital readmission rates (0.6 ± 0.9 vs 0.2 ± 0.6) [29]. These findings indicate that, in the absence of adequate preoperative optimization and vascular management, surgeons may reasonably favor a more proximal level to ensure healing. Nevertheless, the functional consequences of this choice are clinically relevant: a meta-analysis conducted on 27 studies showed that preserving the knee joint is consistently associated with superior functional outcomes, with a substantially higher proportion of patients undergoing BKA being able to ambulate independently for distances of 500 meters or more compared with those undergoing AKA [30]. Taken together, these observations suggest that the predominance of AKAs in our study likely reflects not only patient frailty and disease severity, but also potential gaps in the organization and effectiveness of PAD care pathways, highlighting the need for more homogeneous access to timely diagnosis, revascularization, and multidisciplinary limb-salvage strategies across the SSN.

Over 2015–2021, the cumulative SSN hospital expenditure associated with PAD-attributable major amputations was €294 million, with AKAs contributing €221.8 million and BKAs €72.2 million. Total inpatient days were 511,790 (AKAs: 372,145; BKAs: 139,645). Mean expenditure per HD was similar between levels (€11,115 for AKAs; €11,182 for BKAs), but BKAs had longer mean LOS (22 vs 19 days). Our finding of a longer LOS for BKAs compared with AKAs is consistent with evidence from a large Danish nationwide registry study that examined temporal trends in LOS by initial amputation level between 2010 and 2021. When restricting the comparison to the period overlapping with our analysis (2015–2021), median total LOS values in the Danish study were consistently lower than those observed in our cohort, yet preserved the same relative pattern by amputation level. Specifically, during 2015–2021, median total LOS for transtibial amputations declined from 20 days in 2015 to 14 days in 2021, whereas corresponding values for transfemoral amputations decreased from 14 days to 11 days [31]. The longer BKA stay is clinically plausible: BKAs are often pursued when limb salvage has been attempted or when there is a goal of preserving knee function, which can translate into more complex perioperative management, staged procedures, infectious disease management, wound care needs, and discharge planning [32]. With regard to the costs associated to amputations, very few studies have quantified this burden, especially in the European context. A German study estimated hospital-related expenditures exceeding €16,000 during the week of amputation [33]. A meta-analytic review of diabetes-related complication quantified costs across six industrialized countries (Australia, Canada, France, Germany, Italy, and Spain), based largely on DRG tariffs and standardized to 2003 values, estimating a hospital expenditure of €10,177 for lower-limb amputation in Italy, among the lowest reported internationally (ranging from €14,787 in Spain to €31,998 in France) [34].

Consistent with our longitudinal analysis, previous studies have shown that patients undergoing BKA tend to be younger and more frequently male compared with those undergoing AKA [31,35,36]. During the two-year baseline period preceding the index amputation, prior revascularization was more frequent in BKA patients (57.5%) compared with AKA patients (42.2%), with consistently higher proportions among diabetic patients. Only 14.4% of patients had revascularization during the index event, and only 16.1% of patients had a minor amputation together with revascularization during baseline. Although international guidelines identify revascularization as a cornerstone intervention for preventing amputation in patients with PAD and CLTI [37], its utilization rate in the study population was found to be remarkably low. The higher prevalence of prior revascularization among BKA patients suggests that timely vascular interventions may increase the likelihood of achieving a transtibial rather than a transfemoral amputation, which is generally considered a more favorable functional outcome. A German cohort study conducted between 2013 and 2015 showed that 81.8% of patients underwent at least one vascular diagnostic procedure and 61.0% received a vascular surgical intervention prior to lower-extremity amputation [38]. Analysis of the interval between the last revascularization and the index amputation showed that, among patients who underwent revascularization during the baseline period, a substantial proportion experienced a prolonged gap before amputation, with 23% of those undergoing AKA and 15% of those undergoing BKA having their last revascularization more than six months prior to the index event.

Beyond the mere prevalence of revascularization, the timing of the last vascular intervention before amputation appears clinically relevant. If a patient has undergone revascularization only a few weeks earlier, a repeat attempt at limb salvage may be less likely to be pursued, whereas a longer interval since the last procedure could reasonably justify reconsideration of revascularization before proceeding to major amputation. In this context, our findings show that among revascularized patients, the last intervention occurred 57 days before the index event in 50% of AKA patients and 40 days before the event in 50% of BKA patients. Moreover, BKA patients not only showed higher rates of prior revascularization, but also a more favorable timing of these procedures, with interventions occurring closer to the index event. The timely vascular management may represent an additional determinant contributing to the higher likelihood of achieving a transtibial rather than transfemoral amputation, and ultimately to better functional outcomes. In line with our findings on timing of prior revascularization, a study in diabetic patients with critical limb ischemia showed that clinical recurrence of ischemia after percutaneous transluminal angioplasty occurred in 24% of cases within a mean of 3.5 months and was associated with markedly higher rates of major amputation (24% vs 4%) and death (33% vs 8%) [39].

A positive finding is the increase over time in the proportion of amputation-related HDs that also included a revascularization procedure, particularly for BKAs (from 16.5% in 2015 to 22.7% in 2021). Data from a Danish national registry indicate a substantial decline in major amputation incidence, decreasing from 41.67 to 32.53 per 100,000 person-years when comparing the periods 1997–2002 and 2009–2014. Over the same timeframe, the proportion of patients referred for evaluation by vascular surgeons rose steadily, increasing from 25.7% to 31.3% [40].

Gangrene diagnosis during baseline was extremely frequent across groups (overall 89.4%), peaking in diabetic BKA patients (93.2%). The high proportion of patients diagnosed with gangrene is expected, as PAD and diabetes are among the main risk factors for the development of gangrene [1,41]. Baseline inpatient utilization was relevant: mean cumulative LOS during baseline was 33.7 days per patient overall, markedly higher in diabetic patients (e.g., AKA with diabetes 39.6 days vs AKA without diabetes 24.9; BKA with diabetes 40.4 vs BKA without diabetes 30.5). These findings suggest that most patients arrive to major amputation after a prolonged and resource-intensive disease course characterized by advanced tissue loss and repeated HDs—consistent with the natural history of CLTI in high-risk populations (especially diabetes). They also support the interpretation that major amputation frequently represents an endpoint after prior attempts at limb salvage, rather than a first-line approach. The longer baseline LOS observed in BKA compared with AKA may reflect more complex inpatient management and repeated interventions aimed at preserving the knee joint before proceeding to definitive amputation. A UK study assessing inpatient resource utilization in patients with CLTI reported substantial hospital consumption across revascularization procedures, with median lengths of stay of 15 days for bypass surgery, 6 days for endarterectomy, 7 days for angioplasty, and 11 days for thrombo-embolectomy [42].

The age- and sex-adjusted Cox estimates showed very high in-hospital mortality, with a steep rise early after amputation. At 180 days, mortality ranged from 16% to 25% across strata; during the index hospitalization alone, mortality ranged from 8% (BKA non-diabetes) to 13% (AKA diabetes). By 730 days, in-hospital mortality reached 34% in AKA with diabetes, 30% in AKA without diabetes, 25% in BKA with diabetes, and 23% in BKA without diabetes. In an Italian cohort study of patients with diabetes enrolled between 2012 and 2015, mortality after major lower-limb amputation reached 33% at one year and increased to 65% at four years [22]. These estimates are substantially higher than those observed in our analysis, as they capture all-cause mortality, including deaths occurring outside the hospital setting. Nevertheless, even when restricting the assessment to in-hospital mortality, our study was able to capture a considerable proportion of the overall mortality burden associated with major lower-limb amputation. The same study confirmed diabetes is a major predictor of mortality after amputation, while others confirmed a higher short-term mortality associated with AKA compared with BKA, reporting 30-day mortality rates of 12.7–21.7% for AKA versus 6.2–17.4% for BKA [43]. In this context, the high prevalence of AKAs observed in our cohort is not only relevant from a functional perspective, but also carries important prognostic implications, given the consistently higher mortality associated with transfemoral compared with transtibial amputation.

Consistent with our results, a recent US Medicare study demonstrated that a higher intensity of vascular care in the year preceding major amputation was associated with better outcomes: patients receiving high-intensity vascular care had a significantly lower risk of all-cause mortality at two years after amputation (HR 0.79; 95% CI 0.74–0.85), highlighting the prognostic importance of adequate pre-amputation cardiovascular care [44].

Among the 1,281 deaths during the index hospitalization, 72.8% had CVD as the principal diagnosis and 92.6% had CVD as either principal or secondary diagnosis. Over the entire two-year follow-up (including the index admission), 3,399 deaths were recorded, with 45.2% CVD as principal diagnosis and 79.1% CVD as principal or secondary diagnosis. The high proportion of cardiovascular-related mortality observed during the index hospitalization may be partly influenced by our selection of admissions classified under DRG 113; nevertheless, in the short term, cardiovascular mortality represents one of the leading causes of death after major lower-limb amputation, together with postoperative complications such as wound infections (including bacterial and fungal infections) and thromboembolic events, including deep vein thrombosis and pulmonary embolism, particularly in patients experiencing reduced mobility after AKA [45–47]. Long-term mortality after major lower-limb amputations has been proven to be associated with ASA grade, severe renal disease and increased age (especially considering most of our patients were over 70 years old at index event) [48].

Some limitations must be acknowledged. The study is based on the national SDO, an administrative dataset originally designed for purposes other than epidemiological research. Despite this, the database is well suited for identifying the population of interest, as it is reasonable to assume that all acute major lower-limb amputations are performed in a hospital setting. However, coding practices may lead to an underestimation of certain comorbid conditions, such as diabetes. In general, administrative records lack clinical details like laboratory values or lifestyle risk factors, and they capture mortality only during the hospitalization. Consequently, our outcome analysis includes in-hospital deaths but cannot account for post-discharge mortality, likely underestimating the true total mortality following amputation.

Moreover, our cost assessment was limited to inpatient hospital expenditures. For patients undergoing major lower-limb amputation, a substantial share of the economic and social burden lies outside the hospital setting. Patients and families often face considerable unreimbursed expenses related to prosthetic devices, long-term rehabilitation and physiotherapy, adaptations to the home environment or personal vehicles, and both formal and informal caregiving [49]. Beyond these tangible costs, limb loss is frequently associated with a profound psychological toll [50]. Taken together, these economic and psychosocial stressors may contribute to worsening financial vulnerability and adversely affect clinical trajectories, increasing the likelihood of physical deconditioning, progression to more proximal amputations, higher disease-related morbidity, and premature mortality. A study estimated hospital costs for major amputation that were comparable to those observed in our analysis (approximately $10,639) [51]. However, these inpatient costs were accompanied by substantial additional expenditures, including about $7,151 for prosthetic devices, as well as further costs related to other healthcare services ranging from relatively modest amounts, such as $341 for hemodialysis sessions, to much higher expenditures, reaching up to $55,385 for the management of diabetic foot ulcers. Results from a US study on diabetic patients undergoing a major lower-limb amputation showed that outpatient and pharmaceutical expenditures added a further layer of cost beyond hospitalization, with mean outpatient costs of approximately USD 3,862 and drug costs of USD 3,626 among BKA patients, and comparable outpatient spending (USD 3,421) but slightly higher pharmacy costs (USD 3,953) among those undergoing AKA [19]. Lower-limb amputation is also associated with substantial indirect costs, as it often leads to a marked loss of patient productivity and a long-term reliance on formal or informal caregivers, imposing additional economic and social burdens on families and society [49,52]. Finally, in cases where multiple amputation procedures were coded within the same hospitalization, we classified patients according to the most severe amputation level. This approach may potentially lead to an underestimation of BKA. However, in our cohort only 2.0% of patients had both below- and above-knee amputations coded during the index event (result not shown), suggesting that the magnitude of this potential bias is limited. Despite these limitations, this study represents, to our knowledge, the first nationwide analysis to comprehensively quantify the clinical and hospital-related economic burden of PAD-attributable major lower-limb amputations in Italy, providing population-level evidence on incidence, resource utilization, costs, revascularization patterns, and in-hospital mortality within the Italian National Health Service.

## Conclusions

This study provides a nationwide estimate of the clinical and hospital-related economic burden of major lower-limb amputations attributable to peripheral artery disease in Italy. The findings underscore that major amputation remains a frequent and severe outcome, associated with substantial hospital resource use and high in-hospital mortality. The low rates of revascularization prior to amputation, as well as the high proportion of patients undergoing AKA, suggest missed opportunities for limb salvage and suboptimal adherence to guideline-recommended care pathways.

These results reinforce the need for improved adherence to guideline-recommended management of PAD, with particular emphasis on early diagnosis, timely referral, and appropriate use of revascularization strategies aimed at limb preservation. Although hospitalizations resulting in BKA are associated with longer lengths of stay, this increased resource use is more than offset by significantly lower in-hospital mortality compared with AKA, highlighting the overall clinical value of limb-preserving strategies. Strengthening preventive and multidisciplinary care pathways has the potential to reduce avoidable amputations and mitigate their clinical and economic impact on the Italian National Health Service.

## Supporting information

S1 File Data Figures 1 to 5. (XLSX)
